# The role of mitochondrial dysfunction in the pathogenesis of atherosclerosis: A new exploration from bioinformatics analysis

**DOI:** 10.1097/MD.0000000000042601

**Published:** 2025-05-30

**Authors:** Qiao Fu, Sijie Zhang

**Affiliations:** a Cardiovascular Ward, Department of Geratology, the First Hospital of China Medical University, Shenyang, China.

**Keywords:** atherosclerosis, bioinformatics analysis, drug prediction, gene expression, machine learning, mitochondrial dysfunction

## Abstract

Atherosclerosis (AS) is a complex cardiovascular disease associated with mitochondrial dysfunction (MD), which contributes to plaque formation and instability. This study explores the relationship and shared risk factors between the pathogenesis of AS and MD, aiming to advance preventive and therapeutic strategies for these comorbidities. Data from GSE28829, which includes 13 early and 16 advanced atherosclerotic plaque samples from human carotid arteries, were retrieved from the Gene Expression Omnibus database. Mitochondrial-related genes were sourced from the MitoCarta3.0 dataset. Differentially expressed genes were identified using the “limma R” package in R Studio. A gene co-expression network was constructed using the GeneMANIA database, and gene ontology and Kyoto Encyclopedia of Genes and Genomes pathway analysis were conducted using “clusterProfiler” R package. Candidate co-feature genes were identified using least absolute shrinkage and selection operator, random forest and support vector machine methods. Single sample gene set enrichment analysis on co-feature genes, and co-expression patterns and differential expression were visualized. Drug-protein interactions were predicted using the Drug Signature database, and molecular docking was used to select stable structures. A total of 571 differentially expressed genes and 15 interacting genes were obtained. Gene ontology functional enrichment primarily focused on pathways such as nuclear division and mitotic nuclear division, whereas Kyoto Encyclopedia of Genes and Genomes functional enrichment primarily focused on cell cycle, cellular senescence, and oocyte meiosis. 4 co-feature genes – *ALDH1B1*, *CRY1*, *EFHD1*, and *NIPSNAP3B* – were identified as potential diagnostic biomarkers using least absolute shrinkage and selection operator, random forest and support vector machine methods. These genes influence AS development through various biological pathways, with significant differences noted in pathways such as KRAS. 3 potential drugs, ISOSORBIDE DINITRATE, Benzaldehyde, and Hydroperoxycycloofosfamide – were identified as interacting with ALDH1B1, with interactions verified through molecular docking. This study demonstrates the relationship between AS and MD through bioinformatics and machine learning, identifying 4 key diagnostic genes and potential therapeutic drugs. These findings suggest avenues for new AS treatments. Future experimental validation further exploration AS–MD interactions and MD-based therapies are warranted.

**Highlights**

1. This study demonstrated the relationship between AS and MD through bioinformatics and machine learning, suggesting that MD plays a crucial role in the occurrence and development of AS.
2. This study identified key diagnostic genes, ALDH1B1, CRY1, EFHD1, and NIPSNAP3B using Machine learning. The differential expression of these co-feature genes is related to AS severity and is involved in multiple biological pathways, such as the cell cycle, cell aging, and MD.
3. This study predicted 3 drugs associated with ALDH1B1: ISOSORBIDE DINITRATE, Benzaldehyde, and Hydroperoxycycloofosfamide, verified their protein interactions with ALDH1B1 through molecular docking, providing new ideas for the treatment of AS.

## 1. Introduction

Atherosclerosis (AS) is a complex cardiovascular disease, the pathogenesis of which involves various cellular and molecular mechanisms.^[1]^ In recent years, an increasing number of studies have demonstrated that mitochondria play a key role in the onset and progression of AS.^[2,3]^ Mitochondria are the energy factories of cells, responsible for the production and metabolism of energy within the cell.^[4]^ However, mitochondrial dysfunction (MD) can lead to increased intracellular oxidative stress, enhanced inflammatory responses, and accelerated apoptosis, all of which are closely related to the pathological process of AS.^[5–7]^

MD promotes the formation and instability of atherosclerotic plaque.^[8]^ First, MD leads to increased intracellular oxidative stress, which damages vascular endothelial cells, promotes the recruitment and activation of inflammatory cells, and accelerates the progression of atherosclerotic plaques.^[9,10]^ Second, MD enhances the inflammatory response of cells, and the overexpression of inflammatory cytokines can promote the instability of atherosclerotic plaque.^[11]^ Additionally, MD promotes apoptosis, and increased apoptosis can lead to the rupture of atherosclerotic plaques, resulting in acute cardiovascular events.^[12]^

Therefore, we used gene databases to integrate and analyze gene data related to the pathogenesis of AS and MD, explore the pathogenesis of AS in the context of MD, understand their shared risk factors, and promote the development of preventive measures and treatment strategies for these 2 comorbidities.

## 2. Methods

### 2.1. Data extraction

GSE28829, which contains 13 early atherosclerotic plaque samples and 16 advanced atherosclerotic plaque samples from human carotid arteries, was obtained from the Gene Expression Omnibus database (https://www.ncbi.nlm.nih.gov/geo/query/acc.cgi) using the Affymetrix GPL17077 platform and Affymetrix Human Genome U133Plus2.0 Array.

A total of 1136 human mitochondria-related genes (MRGs) (Table S1, Supplemental Digital Content, https://links.lww.com/MD/P39) were obtained from the MitoCarta3.0 dataset (https://www.broadinstitute.org/mitocarta).

### 2.2. Identification of differentially expressed genes (DEGs)

Differential expression analysis of GSE28829 was performed using the R package Limma, and visualized using the ggplot2 package. Genes with an adjusted *P*-value < .05 and | logFC (fold change) | ≥ 0.585 were identified as DEGs. A gene expression volcano plot and heatmaps were created, and the top 20 DEGs with the most significant differential expression were visualized using the Xian Tao Academic Platform (https://www.xiantaozi.com/). The DEGs and MRGs were intersected to identify AS and mitochondrial intersection genes.

### 2.3. Protein–protein interaction analysis

GeneMANIA (https://www.genemania.org/) was used to construct a co-expression network of the intersecting genes to identify their internal associations within gene sets.

### 2.4. Gene functional enrichment analysis

To explore the biological functions and pathways of intersection genes, we used the “clusterProfile” package in R software and conducted gene ontology and Kyoto Encyclopedia of Genes and Genomes analyses with a *P*-value of .05. We used Xiantao Academic Platform (https://www.xiantaozi.com/)to visualize the results.

### 2.5. Identification of co-feature genes using machine-learning algorithms

The “glmnet,” “randomforest,” and “svmRFE” packages of R software for least absolute shrinkage and selection operator (LASSO), random forest (RF) and support vector machine (SVM) analysis, respectively,^[13,14]^ to further investigate potential co-feature genes related to the diagnosis of AS and mitochondria.

For LASSO λ selection, we used 10-fold cross-validation. We set the λ value within a relatively large range and then used cross-validation to calculate the model error corresponding to each λ value. Finally, we selected the λ value with the smallest transfer error as the final parameter. We also use 10-fold cross-validation to determine the number of trees in a RF. We tried different numbers of trees, such as 50, 100, and 200, and compared their performances, then selected the number of trees (100) that performed the best. SVM-Recursive Feature Elimination model uses a recursive feature elimination process to gradually remove irrelevant or redundant features based on their importance, and determine the optimal feature subset.

Candidate co-feature genes were identified at the intersection of the 3 results.

### 2.6. Single sample gene set enrichment analysis (ssGSEA)

We performed ssGSEA to investigate the effects of co-feature gene expression on the AS pathway.^[15]^ We detected the enrichment of co-feature genes in the gene sets using the clusterProfiler package (v4.4.4), calculated scores through the “GSVA” and “GSEABase” packages, and visualized the association between co-feature genes and the 50 marker gene sets. We constructed the co-expression patterns heatmap and differential expression map of co-feature genes via “corrplot” and “limma” packages.

### 2.7. Drug prediction and molecular docking with co-feature genes

We used Drug signature database (https://dsigdb.tanlab.org/DSigDBv1.0/) to download the relationship files of all drugs and co-feature genes, the Enrichr platform provides access to Drug Signature database. We then sorted the adjusted *P*-values in ascending order and selected the final candidate drugs with the smallest *P*-values.

Molecular docking of the candidate drugs with co-feature genes: We used the PubChem database (https://pubchem.ncbi.nlm.nih.gov/) to search for the compound structure of the final candidate drugs and the PDB database (https://www.rcsb.org/) to search for the structure of co-feature genes. Molecular docking was performed using the CB-DOCK2 website (https://cadd.labshare.cn/cb-dock2/index.Php), and the appropriate drug-gene docking stable structures were selected based on the magnitude of the free energy.

## 3. Results

### 3.1. Identification of 571 DEGs and 15 intersection genes

**Figure 1.**
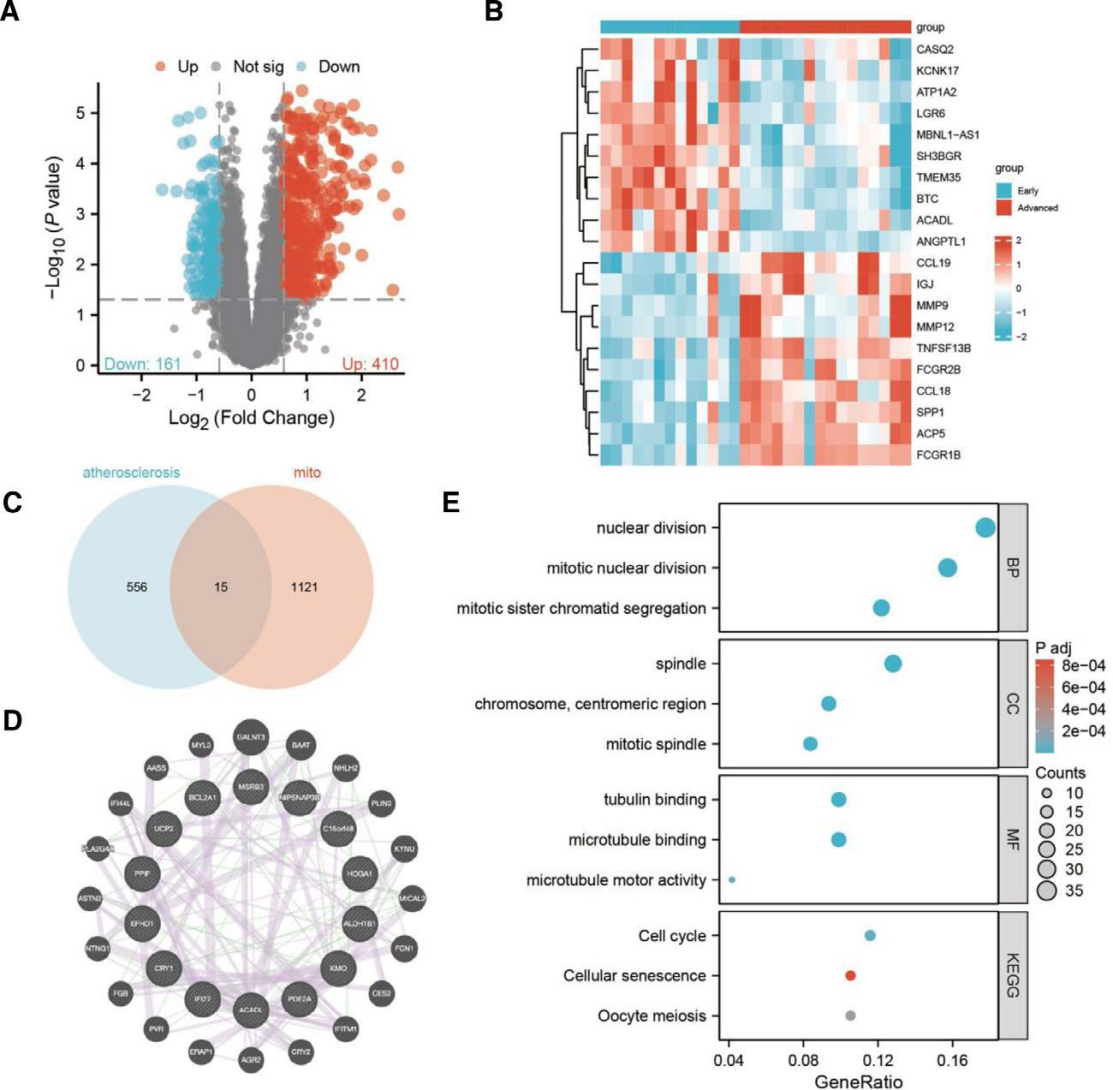
Identification and co- expression network construction of differentially expressed genes (DEGs) related to atherosclerosis (AS) and mitochondrial dysfunction (MD). (A) DEGs with significantly higher (red) and lower (blue) expression levels were showed in volcano plot; (B) top 20 DEGs with the most significant differential expression were showed in heatmap; (C) 15 interaction genes from DEGs and mitochondrial related genes (MRGs); (D) co-expression network between fifteen interaction genes and the 20 most closely related genes. (E) Results of gene ontology (GO) and KEGG (Kyoto Encyclopedia of Genes and Genomes) analyses.

571 DEGs, including 410 and 161 up- and downregulated genes, respectively, were identified from GSE28829. The expression of these DEGs is shown as a volcano plot (Fig. 1A), and the top 20 significantly expressed DEGs are shown in a heatmap (Fig. 1B). 15 intersection genes were identified (Fig. 1C).

### 3.2. Protein–protein interaction network analysis and functional enrichment pathways analysis

A co-expression network was constructed between the fifteen intersection genes and 20 highly correlated genes (Fig. 1D). Gene ontology functional enrichment primarily focused on pathways such as nuclear division and mitotic nuclear division, whereas Kyoto Encyclopedia of Genes and Genomes functional enrichment primarily focused on cell cycle, cellular senescence, and oocyte meiosis (Fig. 1E).

### 3.3. Identification of 4 co-feature genes via LASSO and RF

**Figure 2.**
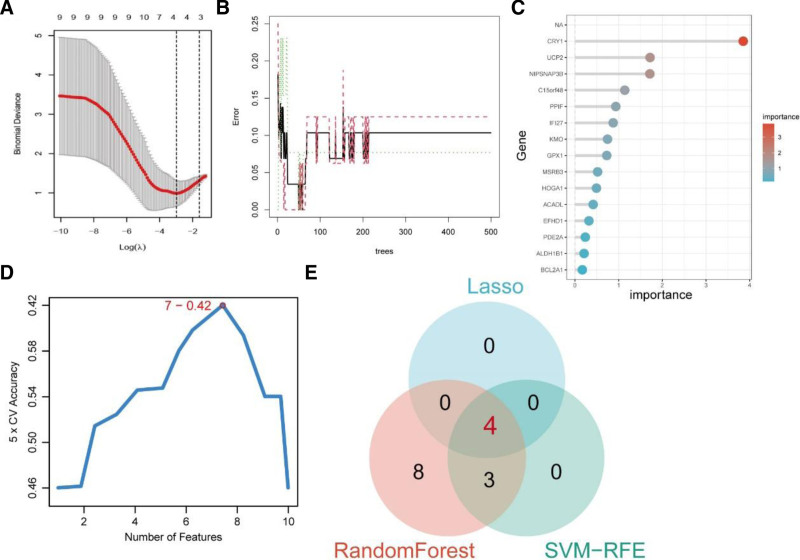
Identification of candidate co-feature genes via machine learning. (A) 4 closely related genes identified via least absolute shrinkage and selection operator analysis (LASSO); (B,C) 15 interaction genes ranked via random forest analysis (RF); (D) result of support vector machine (SVM) analysis; (E) Venn diagram showed 4 co-feature genes, ALDH1B1, CRY1, EFHD1, and NIPSNAP3B were intersected.

The LASSO analysis identified 4 closely related genes (Fig. 2A). RF analysis ranked the importance of the intersection genes based on mean decrease accuracy and mean decrease gini (Fig. 2B, C). And the result of SVM was shown in Figure 2D. 3 results intersected (Fig. 2E), and 4 genes, *ALDH1B1*, *CRY1*, *EFHD1*, and *NIPSNAP3B*, were identified as key co-feature genes.

### 3.4. Analysis of co-feature genes expression levels on AS pathway via GSEA

**Figure 3.**
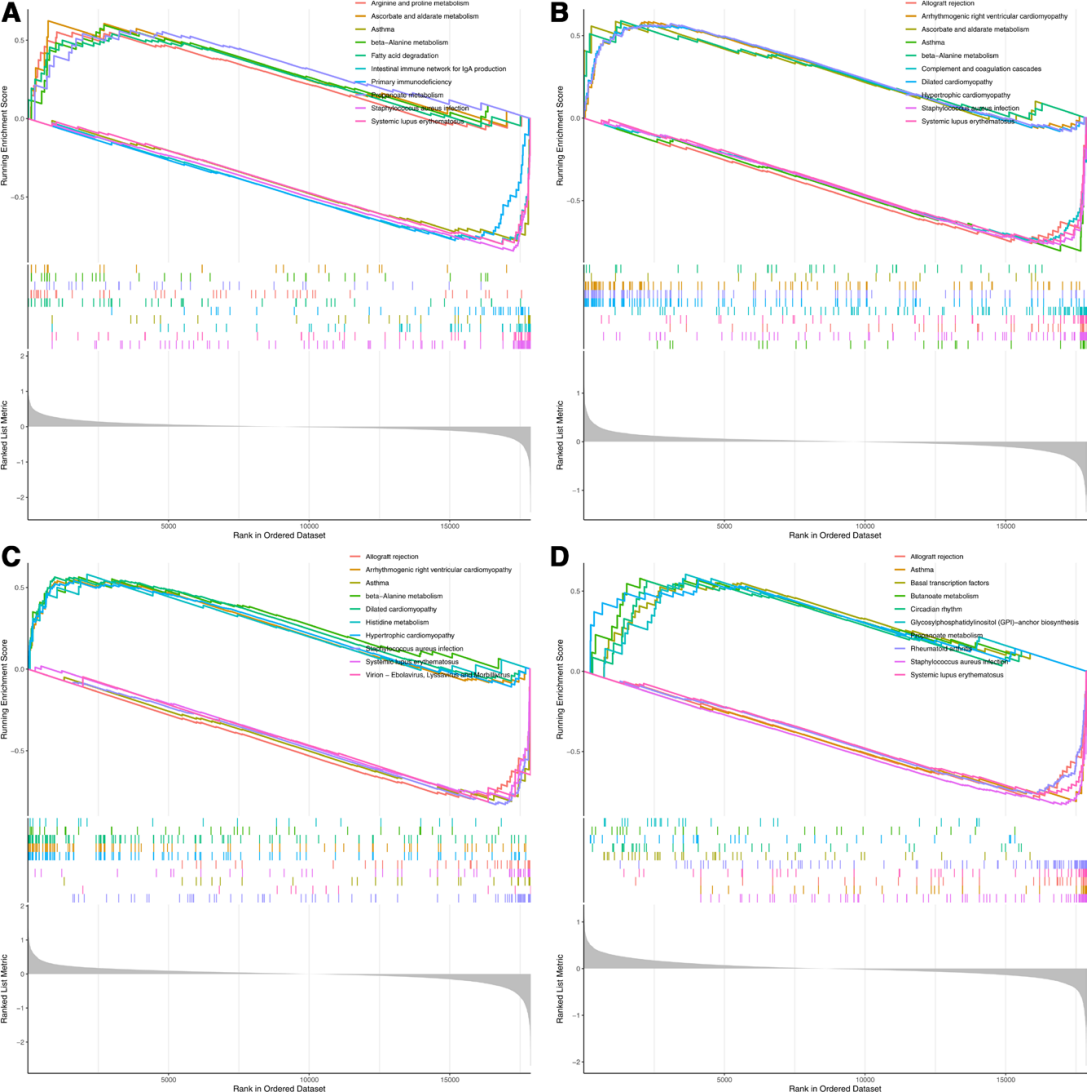
Gene set enrichment analysis (GSEA) results of samples with high and low expression of genes ALDH1B1 (A), CRY1 (B), EFHD1 (C), and NIPSNAP3B (D), and their top 5 enriched pathways of KEGG. KEGG = Kyoto Encyclopedia of Genes and Genomes.

High expression of *ALDH1B1* was enriched in arrhythmogenic right ventricular cardiomyopathy, whereas low expression was enriched in the allograft rejection pathway (Fig. 3A). High expression of *CRY1* was enriched in the arginine and proline metabolism pathways, whereas low expression was enriched in the asthma pathway (Fig. 3B). *EFHD1* high expression was enriched in the arrhythmogenic right ventricular cardiomyopathy pathway, while low expression was enriched in the allograft rejection pathway (Fig. 3C). *NIPSNAP3B* high expression was enriched in the basal transcription factor pathway, while low expression was enriched in the allograft rejection pathway (Fig. 3D).

### 3.5. ssGSEA result of co-feature genes

**Figure 4.**
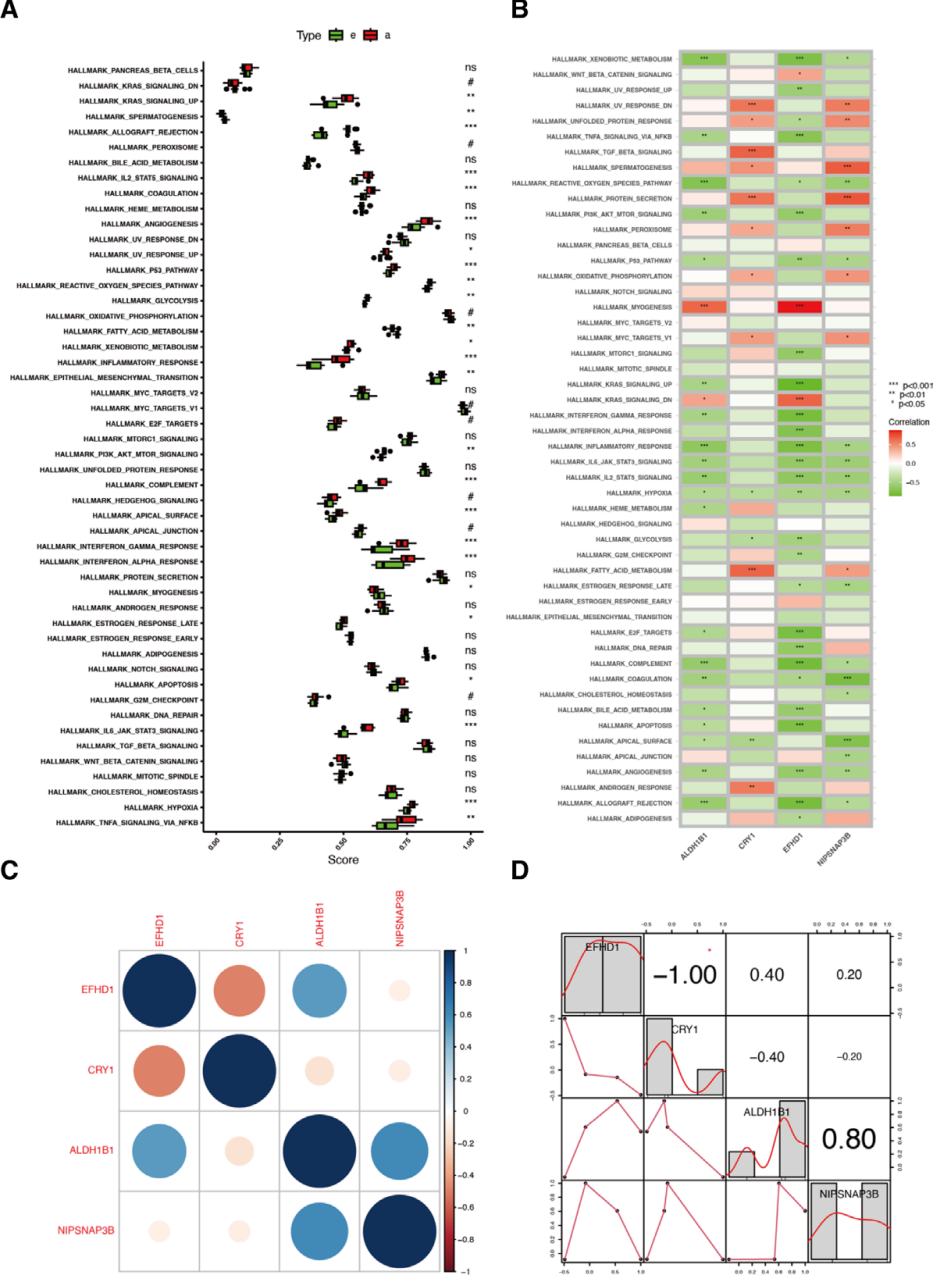
the association between co-feature genes and the 50 marker gene sets. (A,B) Significant differences were observed in pathways such as KRAS from box plot and heatmap; (C,D) the co-feature genes co-expression patterns. *P < .05, **P < .01, ***P < .001, ****P < .0001, ns =no significant difference.

**Figure 5.**
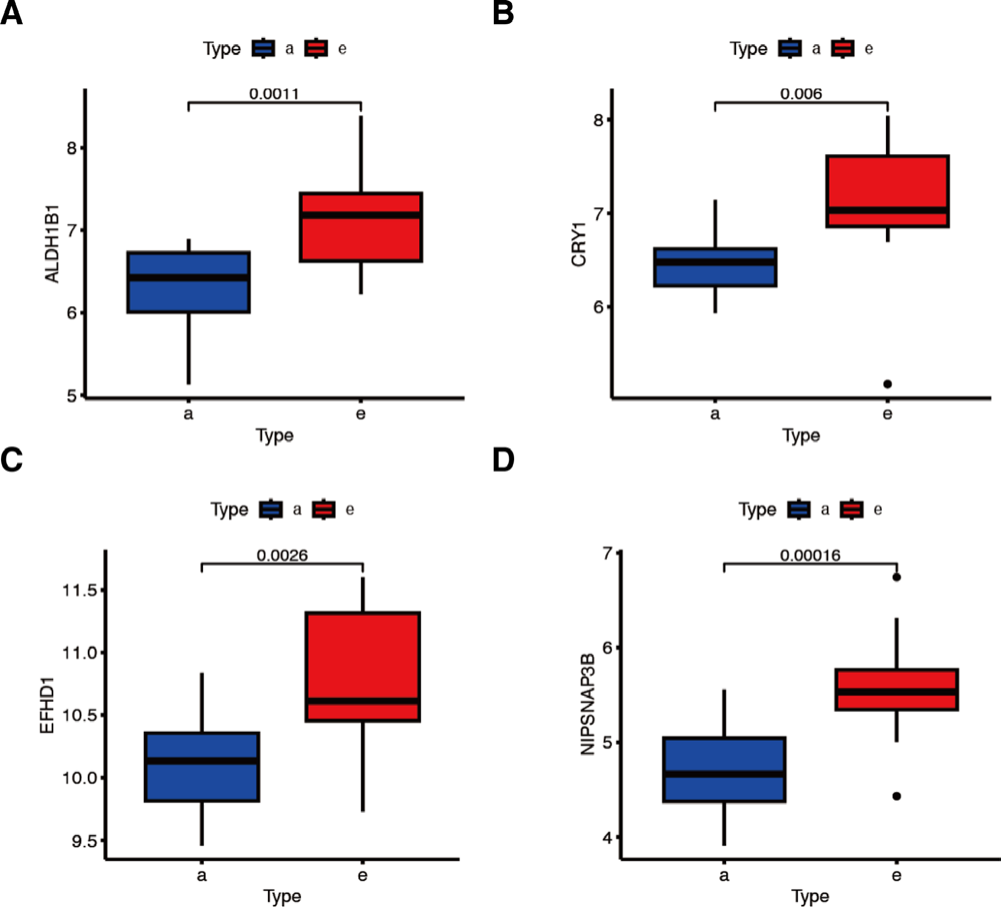
Differential expression of ALDH1B1 (A), CRY1 (B), EFHD1 (C), NIPSNAP3B (D), of early- and advanced-stage AS patients. AS = atherosclerosis.

Significant differences were observed in pathways such as KRAS (Fig. 4A,B). Co-feature gene co-expression patterns were constructed (Fig. 4C,D). In the differential expression map, *ALDH1B1*, *CRY1*, *EFHD1*, and *NIPSNAP3B* were significantly upregulated (Fig. 5A–D).

### 3.6. Identification of potential drugs and construction of stable structures

**Figure 6.**
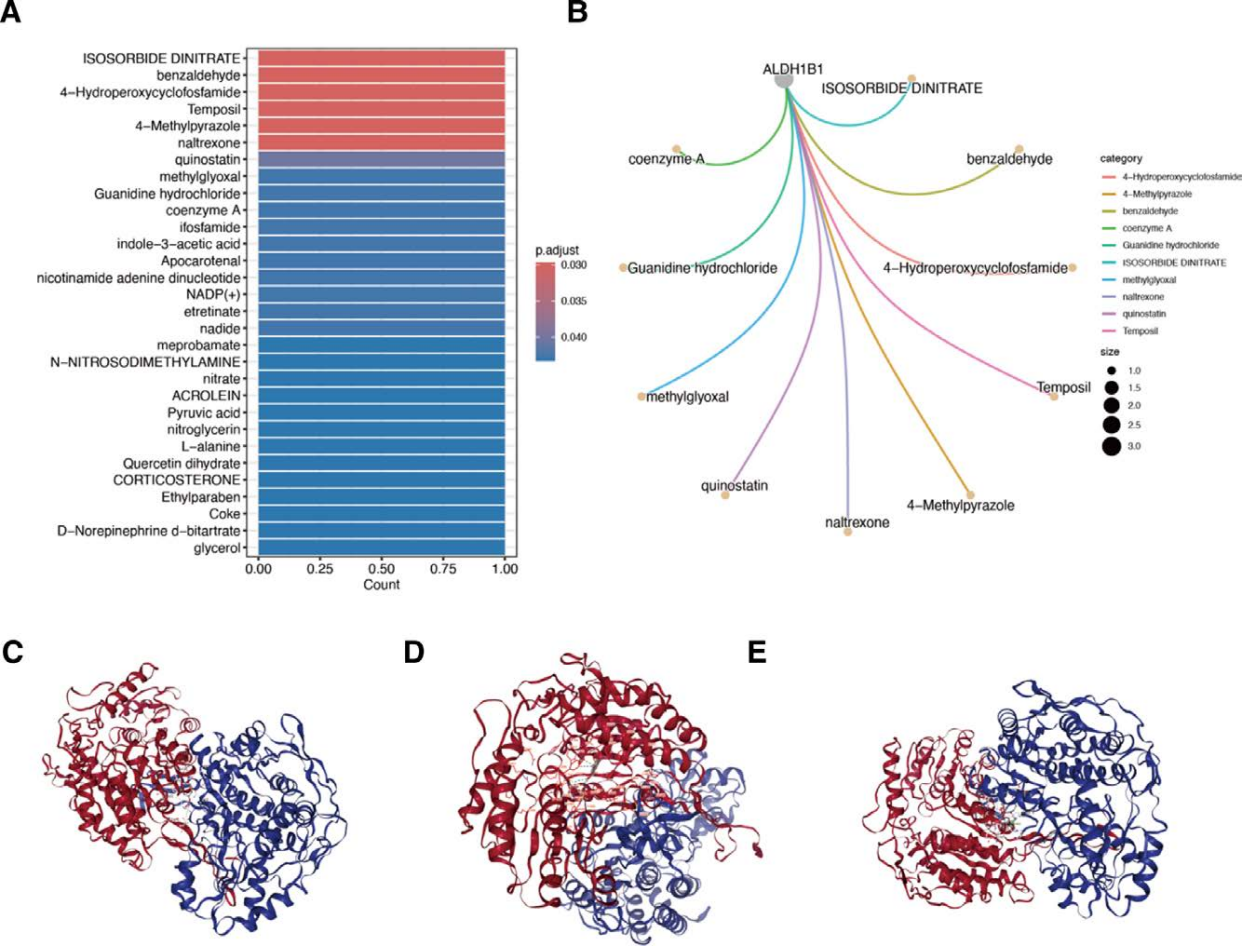
Identification of potential drugs and construction of stable structures. (A) Candidate drugs were sorted in ascending order of adjusted *P*-values; (B) ISOSORBIDE DINITRATE, Benzaldehyde, and Hydroperoxycycloofosfamide, were identified most related to the gene ALDH1B1; (C–E) molecular docking of ALDH1B1 and ISOSORBIDE DINITRATE (C), benzaldehyde (D), Hydroperoxycyclofosfamide (E).

We identified ISOSORBIDE DINITRATE, Benzaldehyde, and Hydroperoxycycloofosfamide, as potentially most closely related to *ALDH1B1* (Fig. 6A,B). Subsequently, we constructed structures of ALDH1B1 with these 3 drugs (Fig. 6C–F).

## 4. Discussion

AS is a prevalent, complex cardiovascular disease. Its pathological progression involves various cellular and molecular mechanisms including inflammation, oxidative stress, abnormal lipid metabolism, and endothelial dysfunction.^[16]^ Research has indicated that MD is a key trigger regulatory factor for AS, significantly accelerating its progression through mechanisms. This study, utilizing bioinformatics analysis and machine learning, revealed a close relationship between AS and MD, identifying key diagnostic genes and potential therapeutic targets. These findings suggest avenues for new prevention strategies and treatment plans.

Our study identified differences in the expression of multiple MRGs in atherosclerotic plaques of patients with AS, and successfully identified key diagnostic genes, *ALDH1B1*, *CRY1*, *EFHD1*, and *NIPSNAP3B* using Machine learning. The differential expression of these co-feature genes is related to AS severity and is involved in multiple biological pathways, such as the cell cycle, cell aging, and MD. This study also predicted 3 drugs associated with ALDH1B1: ISOSORBIDE DINITRATE, Benzaldehyde, and Hydroperoxycycloofosfamide, and verified their protein interactions with *ALDH1B1* through molecular docking, providing new ideas for the treatment of AS.

The expression levels of the 4 key diagnostic genes, *ALDH1B1*, *CRY1*, *EFHD1*, and *NIPSNAP3B*, are downregulated in atherosclerotic plaques of patients with AS, suggesting they may play important roles in the progress of AS and MD. They participate in regulating mitochondrial metabolism, morphology, autophagy, and energy metabolism and affect cell oxidative stress, inflammatory response, and apoptosis, it is consistent with the studies by Duan et al.^[17,18]^ Therefore, these genes are potential diagnostic and therapeutic targets for AS.

Studies have shown that oxidative stress can cause damage to endothelial cells, activate a series of signaling pathways, and affect the expression levels of related genes.^[19]^ *ALDH1B1, CRY1, EFHD1,* and *NIPSP3B*, have partial similarities in structure and function with the gene family involved in oxidative stress response, suggesting that they may be involved in similar intracellular signal transduction processes, regulating the proliferation and migration of vascular smooth muscle cells, which is a key link in the development of AS.^[20]^ Therefore, the expression changes of the key diagnostic genes identified in this study may be potentially associated with the oxidative stress pathway.

From current research, it is known that inflammation plays a central role in the progression of AS, with numerous inflammatory factors such as tumor necrosis factor-α, interleukin-1 (IL-1), and others interacting to form a complex inflammatory regulatory network.^[21]^ Our result indicated that the expressions of *ALDH1B1, CRY1, EFHD1,* and *NIPSP3B*, are induced by certain inflammatory factors, and their expression outcomes may regulate the intensity of inflammatory response by affecting the activity of key proteins in the inflammatory signaling pathway, thereby affecting the occurrence and development of AS.

*ALDH1B1* is primarily expressed in the mitochondrial matrix and participates in fatty acid and ketone body metabolism. It is responsible for converting toxic aldehydes into nontoxic carboxylic acids and protecting mitochondria from oxidative damage.^[22]^ Studies have shown that the deficiency of *ALDH1B1* leads to increased intracellular oxidative stress, enhanced inflammatory response, and accelerated apoptosis, all of which are closely related to the pathological process of AS, suggesting that it may participate in the occurrence and development of AS.^[23–26]^

*CRY1* is a key clock gene regulating circadian rhythms and biological clocks.^[27,28]^ Research has shown that *CRY1* interacts with mitochondrial proteins, influencing mitochondrial function and energy metabolism.^[29]^ Its downregulation suggested it may participate in the occurrence and development of AS.^[30]^ Studies have revealed that the deficiency of *CRY1* can lead to MD, increased oxidative stress, and accelerated cellular aging, all of which are linked to AS.^[31,32]^

*EFHD1* is a mitochondrial protein that regulates mitochondrial morphology and function.^[33,34]^ It inhibits mitochondrial fission and maintains mitochondrial network integrity by interacting with the mitochondrial fission protein Drp1. The expression of *EFHD1* is downregulated suggested it may participate in the occurrence and development of AS. Research has shown that the deficiency of *EFHD1* can lead to abnormal mitochondrial division, mitochondrial network disorders, and accelerated cellular aging, all of which are closely related to the pathological processes of AS.^[35,36]^

*NIPSNAP3B* is involved in mitochondrial autophagy regulation. It interacts with autophagy-related proteins to promote mitochondrial autophagy, clearing damaged mitochondria and maintaining mitochondrial function. Research has shown that the deficiency of *NIPSNAP3B* can lead to impaired mitochondrial autophagy, accumulation of damaged mitochondria, and accelerated cellular aging, all of which are closely related to the pathological processes of AS.

The known AS-related biomarker, *PCSK9*, mainly regulates the degradation of low-density lipoprotein receptor, affects plasma low-density lipoprotein cholesterol levels, and thus participates in the occurrence and development of AS.^[37]^ The 4 genes we identified do not directly affect the low-density lipoprotein receptor metabolic pathway, but indirectly affect lipid deposition and inflammatory microenvironment in the vascular wall by affecting the function of vascular smooth muscle cells. Besides, another known AS-related biomarker, *IL-6,* as an important pro-inflammatory cytokine, it plays a crucial role in the inflammatory cascade of AS, promoting the recruitment and activation of inflammatory cells.^[38]^ The 4 genes identified in this study may affect the responsiveness of smooth muscle cells to inflammatory stimuli by regulating intracellular signal transduction, and together with *IL-6*, participate in the pathological process of AS at different levels.

This study has several limitations. We primarily relied on bioinformatics analysis; more experimental verifications, such as gene elimination and drug intervention, are needed in future research to clarify the functions and mechanisms of key diagnostic genes and potential therapeutic targets.^[39]^ We further revealed an association between AS and MD. However, the specific mechanism of this interaction remains unclear. Further research is needed on how MD affects the occurrence and development of AS, and how AS leads to MD. Finally, we predicted 3 drugs associated with *ALDH1B1* that could be further studied for their therapeutic effects on AS and explored other MD-based AS treatment options in the future.

## 5. Conclusions

This study demonstrates the close relationship between AS and MD through integrated gene analysis and machine learning, identifying 4 key diagnostic genes as potential therapeutic targets. The results provide valuable insights into the pathological mechanisms of AS and the development of new prevention strategies and treatment plans, potentially leading to breakthroughs in the prevention and treatment of AS.

## Author contributions

**Conceptualization:** Qiao Fu.

**Data curation:** Qiao Fu.

**Formal analysis:** Qiao Fu.

**Investigation:** Qiao Fu.

**Methodology:** Sijie Zhang.

**Supervision:** Sijie Zhang.

**Validation:** Sijie Zhang.

**Writing – original draft:** Qiao Fu.

**Writing – review & editing:** Sijie Zhang.

## Supplementary Material


