# Supplementary material for: The role of mitochondrial dysfunction in the pathogenesis of atherosclerosis: A new exploration from bioinformatics analysis
Source: Medicine (Baltimore). 2025 May 30;104(22):e42601. doi: 10.1097/MD.0000000000042601 (PMC12425088; doi:10.1097/MD.0000000000042601)
Supplement: Supplementary file 1 [file medi-104-e42601-s001.pdf]

Table S1 1136 Human Mitochondrial related genes (MRGs) obtained from MitoCarta3.0 dataset.

Gene
CYC1
SDHB
COQ7
SDHA
UQCRC1
COQ5
PDHA1
COQ9
MRPL12
ATP5F1D
COX5A
ISCA2
PMPCB
UQCRFS1
ATP5F1A
OGDH
PDHB
UQCRC2
SDHD
MRPS35
UQCRQ
MRPL53
DBT
PDK4
MDH2
MRPS27
CS
GRPEL1
DLAT
LRPPRC
DLST
PDHX
GFM1
MPC2
NDUFS1
MRPL46
ATP5F1E
SLC25A3
MRPS23
FH

PMPCA
ATP5F1B
SDHAF4
UQCR10
ISCA1
SUCLA2
COQ3
IARS2
MRPS15
IDH3A
COX11
ETFDH
TIMM10
MRPL34
MRPL2
BCKDHA
UQCRH
HIGD2A
ATP5PO
ECHS1
LETMD1
COX6A1
COX15
AFG3L2
HADHA
ETFA
NDUFS7
CPT2
BCKDHB
IDH3B
LARS2
ACADS
LETM1
ATP5ME
OPA1
AUH
SUCLG1
NDUFV2
COQ6
MRPL43
ABHD11
ATP5PF
NDUFB8
LONP1

DLD
AIFM1
ECHDC3
APOOL
MRPL10
MRRF
NDUFS8
ACADM
IMMT
TIMM9
SLC25A4
SAMM50
NDUFS2
NDUFV1
ACO2
SUPV3L1
FECH
MTIF2
PHB
HIBCH
MRPS2
HSPA9
SURF1
PRDX3
GHITM
GUF1
TIMM13
LYRM4
MRPL16
MRPL40
IDH3G
SDHC
NDUFB5
SDHAF2
COQ10A
GATD3A
TXN2
MRPS18A
COX6C
NDUFB9
MTCH2
NDUFA6
SLC25A20
MRPL1

TIMM44
COX17
MICOS10
SOD2
COX6B1
VDAC1
CLPP
HADH
ACADL
ACAT1
MALSU1
CLPX
NDUFS4
MRPL4
C1QBP
PITRM1
UQCC1
MRPL55
MRM1
MECR
MRPL44
HSDL2
MRPS14
ABCB8
ATPAF2
NDUFA9
COA6
POLRMT
ISCU
RTN4IP1
OGDHL
ATP5F1C
BCKDK
GFM2
NDUFAF4
SLC25A35
SLC25A11
TIMM8B
MCAT
IBA57
PHB2
DAP3
CMC2
SDHAF1

MRPS17
ADHFE1
NFS1
NDUFAF5
MRPL17
TACO1
MFN1
PPTC7
MRPL11
COX4I1
NDUFS6
MRPL13
OXCT1
COX5B
PDK2
ALAS1
MRPL33
MTO1
LIAS
NDUFA5
NDUFB6
MTX2
SUCLG2
FDX1
SLC25A1
MRPS28
TIMM50
VDAC2
SLC25A42
TOMM7
ECH1
GATB
BCS1L
ERAL1
CMC1
MRPL28
TSFM
FXN
NFU1
YARS2
MRPL21
ALDH4A1
TOMM40L
NDUFAF1

NDUFB10
ACADSB
MRPS9
COQ8A
ACADVL
COQ4
MRPL15
NDUFA2
MRPL24
HSPD1
NDUFS5
AK3
CYCS
MIPEP
LYRM7
CRAT
PCCB
MRPS7
MRPL3
PRODH
PCCA
MCCC1
MRPS12
CLPB
PDK1
MRPL49
COX7A2
TMEM126A
ECHDC2
HCCS
HIBADH
MRPL19
MRPL36
SLC25A30
BDH1
FARS2
ABCB7
MTX1
NDUFA7
TIMM17A
ALDH9A1
MRPS18C
MARS2
ALDH6A1

FDXR
GATC
TRAP1
ACAD8
ALDH2
PPIF
TIMM22
IVD
L2HGDH
ETHE1
MRPL20
SLC25A5
SLC25A12
MRPS21
TOMM22
ACAA2
MRPL30
DNAJA3
NDUFB2
MRPS34
ETFB
AFG1L
ATP5IF1
ATP5PB
COX7C
CHCHD3
COQ10B
ACSF3
POLDIP2
SLC25A10
SLC25A13
PDK3
ME3
MRPL22
IDH2
GCDH
MRPL47
PPA2
MRPL9
CHCHD10
WARS2
SLC25A19
CBR4
SMDT1

HAGH
COX7A1
MTG1
COX14
NDUFA12
MRPS16
MTERF2
ETFRF1
GPD2
NDUFA10
NDUFC2
SLIRP
MT-ATP6
MT-CO2
TIMM21
NDUFA8
GLS
NDUFB11
DHTKD1
FAHD2A
HSD17B8
HINT2
MRPS5
C6orf136
SPRYD4
LIPT2
DECR1
MRPS26
SLC25A15
NDUFV3
BPHL
STOML2
MSRB2
LACTB
TOMM40
SLC25A25
ADCK5
ACAD10
VWA8
CCDC90B
DNAJC11
ALKBH7
GLDC
MPC1L

THEM4
SSBP1
MRPL27
HSCB
MRPS10
AK4
ATAD3A
MRPL37
PTGES2
TXNRD2
ACSM5
CISD1
MRPL41
MTARC2
MRPS6
MRPL23
SQOR
SCO1
GCAT
MTHFD1L
ECI2
UQCRB
ATP5PD
ATP5MG
CCDC58
MCCC2
MCEE
MOCS1
MMUT
SLC25A51
SPG7
ATP5MC3
ATAD1
OXNAD1
MTG2
DARS2
MRPL51
PC
CLYBL
PNPLA8
MLYCD
PYURF
TIMM17B
MRPL32

SLC25A26
PRODH2
NIT2
FDX2
ME2
TUFM
COX6A2
SLC30A9
TAMM41
TIMM23
RMND1
MFN2
DNLZ
CHCHD4
NDUFAB1
ACSM1
COA5
COQ2
PDSS2
EHHADH
NDUFS3
ABCB10
CKMT2
MRPS25
ACAD9
COX16
FAM210A
ACOT13
CPT1A
DHRS4
PRELID2
CARS2
NIPSNAP2
NDUFB7
MICOS13
GLRX2
MRPS30
SCO2
HSD17B10
NARS2
RPUSD3
SUOX
SARDH
RIDA

COX8A
SPR
MPV17
ZADH2
MRPL18
ALDH1B1
CHCHD1
TIMM8A
VDAC3
SLC25A24
MPC1
SLC25A22
SLC25A31
SLC25A18
TRNT1
IDE
GSTK1
VARS2
HSPE1
MRPL54
MRPL38
MRPL58
MTCH1
TRIAP1
PTCD3
COX10
AKAP1
MRM3
AK2
TMEM70
NOA1
MCUR1
TMEM14C
NDUFC1
MRPL35
NDUFA3
PDSS1
SLC25A16
FMC1
ACSM3
NDUFAF6
UCP1
SFXN1
GLRX5

PTRH1
MRPS11
XPNPEP3
PDP1
ECI1
SLC25A21
SLC25A29
SLC25A45
SDHAF3
PRELID3B
SLC25A39
NDUFB3
REXO2
GATM
TIMM10B
SARS2
CHDH
MRPL57
C8orf82
EARS2
GRHPR
MTFMT
PTCD2
ECHDC1
HARS2
NDUFA4
NDUFA13
NAXE
TMEM11
SELENOO
NAGS
HSD17B4
NDUFAF7
OAT
MRPL50
COX7B
RARS2
OXLD1
LACTB2
NDUFA11
AURKAIP1
CHCHD7
SHMT2
MTHFD2

RMDN1
CPS1
MRPL14
MTRES1
RDH13
TRMT2B
MRPS24
COA3
GSR
ENDOG
BCAT2
TEFM
ACSF2
TRMU
MRPS22
LAP3
GLUD1
HMGCL
FHIT
MRPS33
NUBPL
LYRM2
MPV17L
GADD45GIP1
TFAM
ALDH18A1
OXSM
KYAT3
TARS2
TBRG4
TMEM65
ROMO1
PRDX5
DNAJC15
MAIP1
HIGD1A
C16orf91
RHOT2
SLC25A44
HAO2
OXA1L
SLC25A46
RDH14
ATP5MPL

CA5B
COASY
CISD3
GSTZ1
COX6B2
GLOD4
ACAA1
CROT
PDF
COX19
AMT
PDPR
NLN
BOLA1
ALAS2
GRPEL2
ALDH7A1
HDHD5
GFER
IMMP2L
COX4I2
MRPL39
AADAT
FUNDC2
LYPLA1
PDHA2
MTRF1L
GTPBP3
MTHFD2L
YME1L1
ACOT2
COA8
MMAB
PPOX
ABCD3
SIRT3
LDHD
MRPL45
C12orf65
IMMP1L
POLG
ATPAF1
BLOC1S1
ALDH1L2

COX18
CPOX
NNT
NDUFAF8
ATP23
DCAKD
ATP5MF
SFXN3
AASS
DIABLO
COX8C
PXMP2
AGXT2
GLS2
APOO
ACAD11
C5orf63
HOGA1
TTC19
NGRN
TCAIM
GCSH
SIRT5
GPT2
TOMM6
FAM162A
ABCB6
MACROD1
TST
MT-CO1
MTFR1L
SDR39U1
DMGDH
SLC25A14
PRELID3A
CHCHD5
TOMM70
NIT1
FAHD1
MCU
CYP27A1
MT-ND2
MT-ND4
MT-ND5

DELE1
FASTKD1
MRPS31
ALDH1L1
PTCD1
HADHB
TRIT1
CRLS1
GLYCTK
SLC25A32
AARS2
EXOG
CHCHD2
KMO
SFXN5
ELAC2
GPX1
CPT1B
FIS1
PRELID1
UCP3
SLC25A27
SLC25A40
SLC25A23
HEMK1
SLC25A36
OMA1
LETM2
DHRS1
STOM
METTL5
MSRA
NUDT8
NMNAT3
SLC25A38
MPV17L2
ACLY
ABCD2
FLAD1
LIPT1
DGLUCY
PCK2
NADK2
MPST

NUDT13
ACACA
ABAT
HMGCS2
ALDH5A1
CKMT1A
PISD
NDUFB4
UQCR11
FAM136A
TOMM5
HTRA2
NDUFA1
MT-CYB
GUK1
D2HGDH
OPA3
AKR1B10
PHYH
NDUFAF2
ECSIT
PARL
MRPL42
DNAJC4
PAM16
ALDH3A2
ACSL6
MRS2
ABHD10
HINT3
PXMP4
OSGEPL1
NUDT2
CYB5B
GRSF1
MRPS18B
DNAJC30
CAT
DGUOK
ACOT9
ACSS1
RFK
STARD7
CHPT1

MT-ATP8
TIMMDC1
SPATA19
HTATIP2
AKR7A2
DUT
MT-CO3
DHRS7B
MTRF1
TMEM143
NT5M
RHOT1
NME4
MTHFS
TIMM29
PNPO
MICU2
PCBD2
QRSL1
ADCK1
PRDX6
NIPSNAP1
GARS1
ACSM2A
SLC25A33
LYRM9
RAB24
PARS2
MRPL52
PUS1
GOT2
NAXD
MT-ND3
ACCS
PTRH2
PGS1
OXR1
LYPLAL1
DNAJC28
FPGS
TMEM205
SLC25A53
AIFM3
MGST1

MTFP1
COA4
NIF3L1
PUSL1
SDSL
BAX
MAVS
METTL17
ACACB
ARG2
MTPAP
CCDC51
SUGCT
DNM1L
NRDC
TMEM126B
NSUN4
ACSS3
FAM210B
RBFA
METAP1D
AHCYL1
NIPSNAP3A
MTARC1
FUNDC1
ACSL1
COX20
HSDL1
CBR3
MTERF4
NUDT19
HEBP1
PTPMT1
PGAM5
DTYMK
KARS1
STAR
DNAJC19
OTC
COX7A2L
MTERF3
SFXN2
FASN
PINK1

TMEM177
TXNRD1
THNSL1
FDPS
IDI1
TOP3A
FTMT
LDHAL6B
MTFR1
BCL2L13
TRMT10C
ACOT7
TFB1M
ARF5
MCUB
TWNK
MMADHC
PYCR2
TSTD1
LYRM1
OSBPL1A
RECQL4
PET100
GDAP1
MTIF3
UNG
GLYAT
ISOC2
ATP5MC1
QDPR
COQ8B
GTPBP10
TSPO
FTH1
TRMT1
ADCK2
TSTD3
MRPS36
PDP2
AGPAT5
MCRIP2
UCP2
SLC25A37
SLC25A28

SLC25A48
SLC25A43
SLC25A47
SLC25A41
SLC25A34
EPHX2
NUDT5
MICU1
SOD1
SERHL2
NDUFAF3
BOLA3
OCIAD2
RSAD1
CHCHD6
FASTKD2
PEX11B
TMEM186
PIF1
FABP1
ABCD1
PLPBP
DBI
PNPT1
TOP1MT
RNASEH1
ABCA9
SCP2
NT5DC3
CMC4
LDHB
YBEY
HINT1
MUL1
TK2
AGMAT
CMPK2
BID
ARL2
SYNJ2BP
MFF
DCXR
NUDT9
AMACR

VPS13D
NUDT6
DMAC2
MRM2
BAK1
DHODH
MAOB
MGST3
ACP6
MRPL48
AGXT
CYB5R3
DDX28
PNKD
MICU3
BNIP3
MTX3
MUTYH
GTPBP6
CYP11A1
OCIAD1
METTL8
SMIM20
RPUSD4
SMIM8
NME6
FKBP8
ACSM4
NLRX1
FOXRED1
TFB2M
PPM1K
ATP5MC2
MT-ND1
TAZ
MSRB3
ACOT11
NIPSNAP3B
NTHL1
MARCHF5
SFXN4
TRMT5
SLC25A6
SLC25A52

OGG1
ACOD1
QTRT1
AIFM2
PRDX2
FBXL4
GPAM
DMAC2L
RMDN3
PRXL2A
ATP5MD
PET117
MAOA
C15orf61
POLQ
BCO2
IFI27
CRYZ
PYCR1
TMLHE
TOMM20
YRDC
COA7
DHRS2
MMAA
MIEF1
C15orf48
LIG3
BNIP3L
FASTKD5
PARK7
COA1
COX7B2
MT-ND4L
SIRT4
UQCC2
USP30
PDE12
NME3
DHX30
FASTK
METTL15
CYP24A1
NOCT

PANK2
CA5A
NT5DC2
ATAD3B
TRUB2
PRKACA
COMTD1
CPT1C
C3orf33
MIGA2
SND1
GLUD2
HPDL
ANGEL2
ANTKMT
UQCC3
PRDX4
CDK5RAP1
PAICS
MGARP
HDHD3
CYP11B2
AGPAT4
RCC1L
FAM185A
PMAIP1
TDRKH
SLC8B1
CYP27B1
POLG2
THEM5
BIK
MTERF1
MTFR2
GPX4
TOMM20L
COMT
MYG1
SPATA20
OXCT2
CCDC127
CRY1
ACSM2B
MYO19

MGME1
SEPTIN4
EFHD1
RAB5IF
APEX1
SPTLC2
DMAC1
ATPSCKMT
NEU4
CASP9
AGK
MCL1
DUS2
PLD6
TOMM34
PRORP
PLSCR3
CKMT1B
NDUFB1
ATP5MF-PTCD1
CASP8
BCL2L1
EXD2
ARMC10
MIEF2
THG1L
GOLPH3
PREPL
FASTKD3
CEP89
GPAT2
NBR1
BOK
FKBP10
SETD9
CYP11B1
CSKMT
MIGA1
RPIA
BBC3
BAD
BCL2
BCL2A1
BCL2L2

CASP3
DMPK
DNA2
MT-ND6
PRKN
ALKBH1
PICK1
BCL2L10
AKAP10
STYXL1
NSUN2
ADCY10
SPHK2
NSUN3
PABPC5
PRSS35
PRIMPOL
SERAC1
STX17
TRMT61B
PDE2A
PLGRKT
ETFBKMT
RTL10
BCL2L11
SPIRE1
C2orf69
ARMCX2
ARMCX1
POLB
SNAP29
ARMCX3
ARMCX6
METTL4
SPHKAP
NAT8L
MCCD1
PIGBOS1
HTD2
RP11_469A15.2
